# The use of N-acetylcysteine in the prevention of hangover: a randomized trial

**DOI:** 10.1038/s41598-021-92676-0

**Published:** 2021-06-28

**Authors:** Veronica Coppersmith, Sarah Hudgins, Jill Stoltzfus, Holly Stankewicz

**Affiliations:** grid.449409.4 St. Luke’s University Health Network, 801 Ostrum Street, Bethlehem, PA 18015 USA

**Keywords:** Nutritional supplements, Nausea

## Abstract

Hangovers resulting from alcohol intoxication can lead to adverse effects ranging from generalized discomfort and work-related absenteeism to emergency department visits from patients seeking symptomatic care. The purpose of this study was to evaluate the efficacy of a low dose (600–1800 mg) of N-Acetylcysteine (NAC) vs placebo on mitigating hangover symptoms. This was a randomized, double-blinded, placebo controlled crossover study involving 49 volunteers who consumed beer to obtain a breath alcohol content (BrAC) of 0.1 g/210L. The participants met on two separate occasions at which time they were given either NAC or placebo capsules. Opposing treatments were administered during the second encounter. The morning after the participant’s intoxication and treatment, a Hangover Symptom Scale Questionnaire was administered to determine subjective changes in hangover symptoms. Data was analyzed by self-control, comparing the participant’s hangover symptom severity when using NAC compared to placebo. No significant difference was found in the general distribution of total hangover scores (*P* = .45) (NAC = 10; Placebo = 13). There was also no significant difference found in the general distribution of specific hangover symptoms. However, a significant difference was found in the general distribution of total hangover difference scores based on gender (*P* = .04) (Female − 3.5; Male 2), specifically for nausea (*P* = .05) and weakness (*P* = .03). Although no difference was found in the general hangover scale scores, the study was suggestive of gender specific susceptibility with female participants having improved hangover symptoms after NAC use.

## Introduction

### Background

“The alcohol hangover refers to the combination of mental and physical symptoms, experienced the day after a single episode of heavy drinking, starting when blood alcohol concentration approaches zero”^1^. Hangovers are a nation-wide problem manifesting in an array of negative outcomes affecting society. The pathophysiologic effects on the human body are numerous and still not completely understood. Ethanol and its metabolites have been found to cause issues with the natural physiologic equilibrium of the human body causing many well-known hangover symptoms^2^. Due to these effects, individuals who consume large amounts of alcohol can develop a multitude of unwanted side effects that include: nausea, headache, fatigue, apathy, vomiting, weakness, dizziness, and thirst^1^. Many of these negative effects are defined in the Hangover Symptoms Scale (HSS), which has been previously discussed as a way to evaluate hangover symptoms and is also utilized in this study in an attempt to quantify drug effectiveness^3^.

The negative effects of alcohol-induced hangover have been found to cause workplace absenteeism, decreased performance in both academic and workplace environments as well as a potential financial burden related to accidents when motorized vehicles or when heavy machinery are utilized^4^. Studies related to clinically effective hangover treatments are limited despite the understanding that alleviation of hangover symptoms could have favorable effects on individuals with positive repercussions for society^5^.

In order to understand the adverse effects of alcohol intoxication and hangover, one must first understand the pharmacokinetic process of ethanol degradation and metabolism at the cellular level. In the liver, alcohol dehydrogenase (ADH) breaks down ethanol to acetaldehyde using coenzyme nicotinamide adenine dinucleotide (NAD+), then aldehyde dehydrogenase (ALDH) oxidizes acetaldehyde to acetate. Acetaldehyde is the toxic byproduct of the first oxidative reaction, which causes oxidative stress, as well as can lead to the formation of other toxic byproducts. It is theorized from animal studies that various antioxidants (glutathione, its precursor cysteine, some vitamins) may alleviate some of the oxidative stress by decreasing the formation of toxic protein adducts in the liver^6^. When excessive amounts of ethanol are consumed the liver is unable to effectively complete this process. As glutathione stores diminish, the patient must wait for the liver to make more glutathione to rid the body of the remaining acetaldehyde toxic byproducts, a process that can take 8–24 h^4^. Since N-acetylcysteine(NAC) is a precursor to L-glutathione, it has the potential to decrease oxidative stress on the liver during ethanol degradation as a glutathione donor. The pharmacokinetics of NAC in assisting with the reduction of toxic metabolites has been seen in numerous studies as an antidote for paracetemol overdose^7^. When NAC was given to rats in conjunction with alcohol, one study noted an increase in glutathione levels. A separate study found that rats pretreated with NAC prior to ethanol ingestion had decreased oxidative stress on the liver and that NAC provided a hepatic protective effect^8^.

The purpose of this study was to investigate the effectiveness of using oral capsules of NAC in an attempt to provide the liver with the necessary coenzymes to decrease oxidative stress on the liver and diminish the unwanted effects of a hangover.

Once NAC is ingested, it reaches peak levels in approximately 45–60 min, and quickly rids itself from the body with a half-life of 1.3 h^7^. When levels are checked in a healthy individual not experiencing stressors on the system, NAC is found to increase the free cysteine although it leaves the total cysteine and glutathione levels unchanged. However, the levels of cysteine and glutathione were increased in individuals whose systems were stressed by paracetamol overdose, suggesting that NAC increased the amount of cysteine and glutathione when the body requires increased levels of the substrates^7^. As previously demonstrated during Tylenol overdose cases, the pharmacokinetics of NAC as a glutathione donor also have the potential to improve the negative toxic effects of acetaldehyde when excess alcohol is consumed^4^.

NAC at low doses (600–1800 mg) has a relatively safe side effect profile. NAC has been associated with rare serious reactions such as hypersensitivity and bronchospasm as well as common less severe reactions such as nausea and vomiting, malodorous stool and mild allergic reaction^9^. These reactions have been associated with much higher doses of NAC than were utilized in this study. Considering the low risk of side effects with lower doses of NAC, it is proposed that it has the ability to do more benefit than harm using these doses in an individual suffering from a hangover.

One recent study found that L-cysteine reduces symptoms of a hangover including nausea, headache and anxiety^10^. However, when reviewing current treatment strategies for hangover, most interventions rely on lessening symptom-based adverse effects. These interventions currently include oral rehydration, caffeine, ibuprofen, and anti-emetics. There is no standard of care other than symptomatic treatment for hangovers as it is generally treated at home without medical care. Since NAC has the potential to enzymatically prevent some the hepatocellular toxic effects associated with hangover symptoms, this study has the potential to reduce hangover symptoms and alleviate the hangover effects of alcohol consumption. NAC is also commercially available over-the-counter for individuals to take at home so it has the potential to prevent hangover symptoms without requiring the financial burden of higher acuity medical care. The effect of this study could have profound significance as it could lead to improved quality of life for the individual as well as societal improvement if academic and work-related productivity can be improved.

## Methods

This was a double-blinded, placebo-controlled study involving healthy, non-alcoholic, (self-reported) volunteers over the age of 21. This study received hospital (St. Luke’s University Health Network) IRB committee approval and was also registered on clinicaltrials.gov (ID NCT02541422 04/09/2015). Informed consent was obtained from each participant and the study was done in accordance with relevant guidelines. The study was conducted on the grounds of a level 1 trauma center and under direct supervision of sober investigators. The participants were thoroughly screened prior to participation to ensure their safety in regards to both alcohol consumption as well as the use of NAC. Participants were excluded from the study if they suffered from the following diseases: alcohol use disorder, diabetes mellitus, kidney or bladder stones, kidney disease, liver disease, or stomach ulcers. Organ transplant patients, dialysis patients, and patients with alcohol, egg, milk or wheat allergies were also not allowed to participate. Female patients had a urine pregnancy test done to ensure they were not pregnant before consuming alcohol. Volunteers taking the following medications were not able to participate: activated charcoal, ampicillin, carbamazepine, cephaloridine, cloxacillin, methicillin, nitrogylcerine, oxacillin, penicillin G, or quinacillin. The participants were also advised of the few minor side effects to NAC including possible allergic reaction, heartburn, and foul smelling bowel movements. The participants were advised that their voluntary participation may lead to a hangover including mild non-life threatening symptoms such as feeling thirsty or dehydrated, fatigue, headache, nausea, vomiting, feeling weak, difficulty concentrating, photophobia, phonophobia, increased sweating, insomnia, anxiety, feeling depressed, and trembling or shaking.

Prior to starting the study, the investigators used PASS software (version 11) to calculate minimum sample size^11^. General guidelines for new medications consider a 20% reduction to be clinically meaningful^12^, which requires a minimum sample size of 41, at alpha = 0.05 and 90% power. Based on survey responses from a convenience sample of 69 medical professionals (including physicians, nurses, advanced practitioners, medics, etc.), the smallest clinically meaningful reduction in hangover symptoms that would represent effective treatment using NAC was 69.4% (from a maximum score of 52 to 15.9), requiring a minimum sample size of only 4. Therefore, we used the more conservative estimate of a 20% reduction for our sample size, resulting in as minimum of 41 subjects for our study.

On each study day, the volunteers met at the study location with the investigators. After signing informed consent, each participant was provided the same type of beer (12 oz of 5.2% ABV Belgian-style wheat ale). They consumed one 12 oz beer at a time until they reached a breath alcohol content (BrAC) of 0.1 g/210L measured with a hospital breathalyzer (Alco Sensor IV) that had been recently calibrated. To properly assess the number of beers required to attain a BrAC of 0.1 g/210L, the participants were breath analyzed 15 min after they finished each beer, until they reached the goal of 0.1 g/210L + /-− 0.005. The number of beers consumed and the associated BrAC after each beer was recorded for each participant. No participant was coerced to drink more alcohol than they felt comfortable consuming and they were able to withdraw from the study at any time. This part of the protocol was the same as another study done at the same institution^13^. Once the individual reached a BrAC of 0.1 g/210L + /-− 0.005, some vapor rub was applied under the nose to conceal the smell of the capsule from the participant, then the participant was given 1–3 capsules of either 600 mg N-Acetyl-L-Cysteine or placebo (Lactose, NF MEDISCA Lot 123,630/E). The placebo and NAC capsules looked identical. The dose of NAC/placebo given was determined by the amount of alcohol consumed. A participant who consumed 1–3 beers was given one capsule, 4–6 beers was given two capsules, and 7 + beers was given three capsules. This algorithm was proposed using a dosing of NAC that would keep participants well below toxic levels in an attempt to remove unwanted negative side effects.

All participants remained at the study site under direct supervision until their BrAC fell below 0.08 g/210L, the legal level for driving. In addition, sober non-participants drove all study participants home. To ensure additional safety of the participants there were sober healthcare providers on site and hospital security was available, if needed.

In the morning, each participant filled out a Hangover Symptom Scale questionnaire, evaluating each hangover symptom (feeling thirsty or dehydrated, feeling more tired than usual, headache, nauseated, vomited, feeling weak, difficulty concentrating, more sensitive to light and sound than usual, sweating more than usual, had trouble sleeping, feeling anxious, feeling depressed, experienced trembling or shaking) on a 5 point Likert scale with 1 representing “Strongly Disagree” and 5 representing “Strongly Agree”. There was also a VAS scale to assess, in general, how hungover they felt from “Feel like I did not drink” to “So hungover I might die”. This was a self-administered questionnaire that each participant was asked to complete within first 1 h of wakening in the morning after the study day. A random number generator was used to determine Placebo or NAC first and the participant was given the other treatment at their subsequent encounter. The person assigning numbers and treatments kept this information from the study investigators to keep the study double-blinded. The study was conducted over the series of many months, and data was analyzed by self-control comparing the participant’s score on the hangover symptom scale when using NAC compared to placebo.

Since the Slutske scale is technically not a validated way to assess hangover, a subset of five valid items from that scale were used to assess the effect of NAC on hangovers^14^. We then created a total score by summing the 5 scales and compared outcomes based on treatment group and gender with separate Mann Whitney rank sums test due to the skewed distributions, using SPSS version 27 (Armonk, NY: IBM Corp). We reported medians and ranges, with *P* < 0.05 denoting statistical significance.

The data was analyzed by a hospital statistician using the numerical values of each category for hangover classification and compared the treatment data to the control data. This research design allows for comparison between hangover symptoms in those that took NAC (treatment), and those that took placebo (control). Due to non-normally distributed data, separate Wilcoxon signed rank tests were conducted to compare the hangover symptoms between NAC and Placebo. For the gender comparisons with difference scores, data was analyzed with separate Mann Whitney rank sums tests. The primary outcome was hangover symptom scores, with secondary outcomes of headache, nausea, and weakness scores. These were compared between the NAC and the Placebo arms for each participant. The primary analysis was for the whole test population, with additional subgroup analysis to compare gender differences (secondary outcome).

## Results

62 participants were enrolled to accommodate for inevitable non-completion by subjects for various reasons. Some participants were unable to complete the study secondary to scheduling conflicts, one participant was unable to tolerate the amount of beer consumed, and one requested withdrawal from the study. 49 volunteers completed the process, which became the sample size. There were 31 males and 18 females. The participants were predominantly white/Caucasian.

No significant difference was found in the primary outcome of general distribution of total hangover scores; NAC median = 10; Placebo median = 13 (*P* = 0.45). There was also no significant difference found in the secondary outcome of general distribution of specific hangover symptoms including headache with NAC and Placebo both with median = 1 (*P* = 0.93), nausea with median score for NAC = 0, and Placebo = 1 (*P* = 0.11), and weakness with NAC median = 1, and Placebo median = 1 (*P* = 0.72). No significant difference in the general distribution of total hangover scores (NAC = 19%; Placebo = 25% of maximum score hangover) (*P* = 0.45).

**Figure 1:**
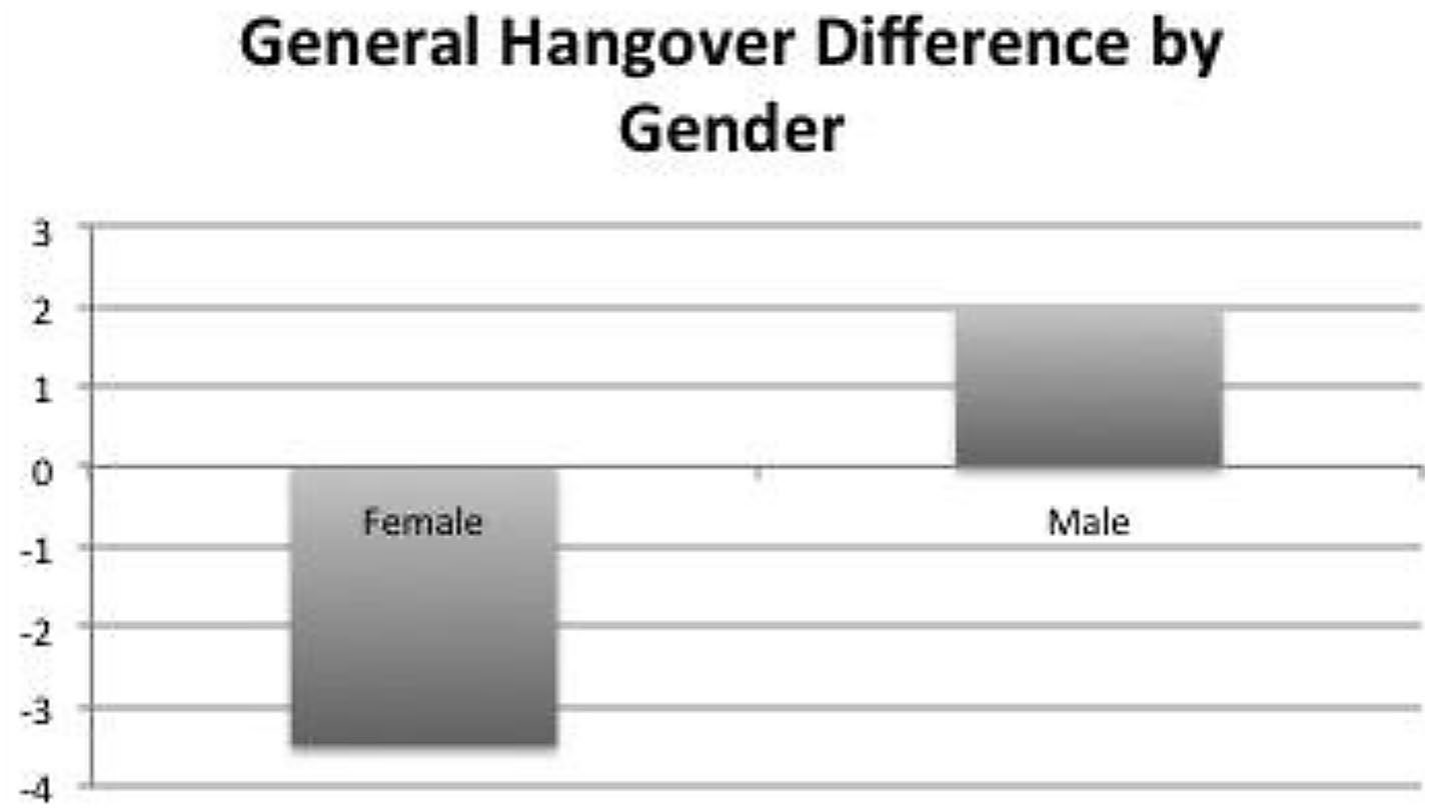
Significant difference found in the general distribution of total hangover difference scores (NAC—Placebo HSS) based on gender (Female − 3.5; Male 2) (*P* = .04), specifically for nausea (*P* = .05) and weakness (*P* = .03). Suggestive of improved HSS NAC vs Placebo in individuals with high EtOH intake and/or high HSS.

There was a statistically significant difference found in the secondary outcome of the total hangover difference (NAC sum—Placebo sum) based on gender, with the female hangover difference of − 3.5, and the male hangover difference of + 2 (*P* = 0.04). (See Fig. 1). Particularly, there were differences found in the important hangover categories of nausea and weakness. There was a significant gender difference for nausea (female difference score range − 4 to + 1, and male range − 4 to + 3) (*P* = 0.05) and weakness (female difference score range − 3 to + 1, and male range − 4 to + 4) (*P* = 0.03). There was no significant difference found between genders for headaches (female difference score range − 4 to + 3, and male range − 3 to + 4) (*P* = 0.32).

There was no statistically significant difference when comparing the subset of the 5 valid hangover symptoms. (See Table 1).

**Table 1:** No statistically significant difference when comparing the subset of the 5 valid hangover symptoms.

	Total Score (median, range)
NAC (n = 45)	5 (0–19)
Placebo (n = 46)	5.5 (0–20)
*P*-value*	0.86
Female (n = 34)	6 (0–19)
Male (n = 57)	5 (0–2 0)
*P*-value*	0.46

*Based on separate Mann Whitney rank sums tests.

At the conclusion of the study, participants were asked to list side effects that they attributed to the pill that they took. Participants noted side effects with both the NAC and the placebo pills. The side effects reported after NAC use included: gastrointestinal (GI) symptoms (4), headache (2), rash (1), muscle twitches (1) and insomnia (1). Participants in the placebo group also reported headaches (3) and GI symptoms (1).

## Discussion

Alleviating hangovers is an important goal due to the implications on productivity for society. Although no difference was found in the general hangover scale scores, the study was suggestive of a gender difference with females having improved hangover symptoms after NAC use. The exact mechanism or reason for this difference is unknown.

There was also suggestive differences in some specific hangover symptoms, specifically alleviation of nausea (*P* = 0.05) and weakness (*P* = 0.03). However, this is based on a post hoc analysis and would require a different study design to further analyze the specific components of the hangover symptom scale. Even a small reduction in these symptoms would be beneficial given that alcohol intoxication and hangovers are a cause of economic hardship, costing society billions of dollars per year in sick-time and lost productivity. According to the CDC, excessive alcohol intake leads to an economic cost of $249 billion per year^15^, with most of the cost (72%) resulting from decreased productivity in the workplace, and 11% of the cost dedicated to health care expenses related to excessive drinking^16^.

NAC at low doses (600–1800 mg) has a relatively safe side effect profile that is well tolerated by most individuals^9^. The participants in this study did not report any serious adverse side effects. There were very few mild side effects reported by the participants taking NAC in this study. GI symptoms were the most commonly reported and may have been the result of NAC or the alcohol consumed. This suggests that NAC at these doses is well tolerated and likely safe for further studies.

Studies related to alcohol do present unique hurdles including safety concerns, recruitment challenges, and logistical issues. The investigators in this study are in no way promoting binge drinking, however, it must be recognized that some individuals are more prone to hangovers than others, even when consuming smaller amounts of alcohol and alcohol intoxication and hangover symptoms are significant issues in society today.

## Limitations

The symptoms of a hangover are mostly subjective which makes the study of hangovers difficult. The Hangover Symptom Scale is a previously verified scale to evaluate hangover symptoms^3^. However, there are drawbacks to using this in evaluation of NAC’s effectiveness on hangovers. This scale was validated using college students and not tested on the general public. Our study participants mostly ranged from 20 to 30 years of age, which is older than most college students, who usually range from 18 to 22 years old. The survey was also self-administered, so it is possible that although participants were told to do it within one hour of wakening, the investigators were not present when the survey was completed to ensure this.

Another limitation of the study is determining if the appropriate dose of NAC was given. Low dose NAC was chosen in this study because of its safe side effect profile, however, it is possible that these lower doses prevented therapeutic levels of NAC thereby limiting the beneficial effects. This algorithm was proposed using a dosing of NAC that would keep participants well below toxic levels in an attempt to remove unwanted negative side effects.

Another potential limitation in this particular study relates to the timing of alcohol consumption by the participants. Each session took place starting in the early afternoon and participants were required to be sober prior to leaving the study. Due to this stipulation, it is possible that the amount of time participants consumed alcohol was diminished and, as such, the hangover symptoms experienced by the participants the following morning were not as severe as it would have been if they had continued drinking later in the evening. Addionally, participants may have consumed more alcohol upon leaving the study site.

The study was open to all ages of legal drinking age, but the participants were largely young adults so it is impossible to say if NAC would have different effects on older adults. Future studies should be done to look at this variable.

## Conclusion

Although no difference was found in the general hangover scale scores, the study was suggestive of a gender difference with females having improved hangover symptoms after NAC use. The study poses the need for a new study that includes a larger, more diverse study group that utilizes a more natural approach to alcohol ingestion allowing individuals to drink the quantity they desire during a timeline that they choose.
