# Author Correction: The use of N-acetylcysteine in the prevention of hangover: a randomized trial

**DOI:** 10.1038/s41598-021-94669-5

**Published:** 2021-07-21

**Authors:** Veronica Coppersmith, Sarah Hudgins, Jill Stoltzfus, Holly Stankewicz

**Affiliations:** grid.449409.4 St. Luke’s University Health Network, 801 Ostrum Street, Bethlehem, PA 18015 USA

Correction to: *Scientific Reports* https://doi.org/10.1038/s41598-021-92676-0, published online 28 June 2021

The original version of this Article contained errors.

Several sentences in the Introduction were factually inaccurate. This is now corrected and in the Introduction,

“In the liver, alcohol dehydrogenase (ADH) breaks down ethanol to acetaldehyde, a toxic by-product, using coenzymes glutathione and cysteine, which are the limiting reagents in this reaction^6^. When excessive amounts of ethanol are consumed the liver is unable to effectively complete this process. As glutathione stores diminish, the patient must wait for the liver to make more glutathione to rid the body of the remaining acetaldehyde, a process that can take 8–24 h^4^. Since N-acetylcysteine(NAC) is a precursor to L-glutathione, it has the potential to decrease oxidative stress on the liver during ethanol degradation as a glutathione donor.”

now reads

“In the liver, alcohol dehydrogenase (ADH) breaks down ethanol to acetaldehyde using coenzyme nicotinamide adenine dinucleotide (NAD+), then aldehyde dehydrogenase (ALDH) oxidizes acetaldehyde to acetate. Acetaldehyde is the toxic byproduct of the first oxidative reaction, which causes oxidative stress, as well as can lead to the formation of other toxic byproducts. It is theorized from animal studies that various antioxidants (glutathione, its precursor cysteine, some vitamins) may alleviate some of the oxidative stress by decreasing the formation of toxic protein adducts in the liver^6^. When excessive amounts of ethanol are consumed the liver is unable to effectively complete this process. As glutathione stores diminish, the patient must wait for the liver to make more glutathione to rid the body of the remaining acetaldehyde toxic byproducts, a process that can take 8–24 h^4^. Since N-acetylcysteine(NAC) is a precursor to L-glutathione, it has the potential to decrease oxidative stress on the liver during ethanol degradation as a glutathione donor.”

Additionally, Reference 6 in the manuscript has been replaced. The original Reference 6 is listed below for the record:

Roth, K. Chemistry of a hangover-alcohol and its consequences. *Chem. Unserer Zeit* **41**, 46–55 (2011).

The original version of the Article has now been corrected.

